# Draft Genome Sequence of the Fish Pathogen *Yersinia ruckeri* Strain 37551, Serotype O1b, Isolated from Diseased, Vaccinated Atlantic Salmon (*Salmo salar*) in Chile

**DOI:** 10.1128/genomeA.00858-14

**Published:** 2014-08-28

**Authors:** Esteban Navas, Harry Bohle, Patricio Henríquez, Horst Grothusen, Fernando Bustamante, Patricio Bustos, Marcos Mancilla

**Affiliations:** Laboratorio de Diagnóstico y Biotecnología, ADL Diagnostic Chile Ltda., Puerto Montt, Chile

## Abstract

We sequenced the genome of a motile O1b *Yersinia ruckeri* field isolate from Chile, which is causing enteric redmouth disease (ERM) in vaccinated Atlantic salmon (*Salmo salar*). The draft genome has 3,775,486 bp, a G+C content of 47.1%, and is predicted to contain 3,406 coding sequences.

## GENOME ANNOUNCEMENT

*Yersinia ruckeri* is an aquatically relevant, Gram-negative pathogen that causes enteric redmouth disease (ERM) in a wide range of fish, but especially in salmonids. Affected fish, predominantly rainbow trout (*Oncorhynchus mykiss*) fry, exhibit mouth and eye hemorrhages, exophthalmia, petechial hemorrhages in internal organs, enteritis, and without antibiotic treatment the infection turns septicemic with high mortalities (1). The disease was controlled in the 1970s by using immersion vaccines prepared with classical motile O1 “Hagerman” strains (2). However, the appearance and spreading of O1 variants without flagellum (3) caused vaccine failures in European countries (4–6) and the USA (7). The enhancement of protection with the inclusion of such a type of strain into the vaccine formulation was demonstrated (8). The use of those bivalent vaccines was introduced in Chilean fry stocks in 2006, which led to successful prevention and control of the disease. But in 2008, ERM outbreaks in vaccinated Atlantic salmon (*Salmo salar*) occurred, from which *Y. ruckeri* O1b was isolated (9). To date, O1b isolates have been recovered from vaccinated fish during sporadic ERM outbreaks, suggesting a reduced vaccine protection.

The species is divided into 4 O-serotypes (10), including 5 subgroups (two O1 and three O2): O1a (classical virulent strains) and O2b strains cause most outbreaks and have a widespread geographic distribution, with O1a being predominant in reared salmonids (10). At present, there is one genome sequence of *Y. ruckeri* available: the ATCC 29473^T^ O1 “Hagerman” strain from Idaho, USA (11). In order to gain insight into the biology of *Y. ruckeri* O1b, we sequenced the genome of the motile field isolate 37551, isolated in 2013 from vaccinated Atlantic salmon presenting ERM clinical signs in Chile. Sequencing was performed on a library prepared with Nextera using the MiSeq platform (Illumina). The run yielded 6,421,748 reads corresponding to 963 Mb of chromosomal sequence. Plasmids were not found. The mean read length was ~150 bp. The data were assembled using the CLC bio assembler into 75 contigs with a size ranging from 255 bp to 336,480 bp. The resulting draft genome had a size of 3,775,486 bp and a G+C content of 47.1%, which is similar to that of the *Y. ruckeri* reference genome (12). Genome annotation using Blast2Go, tRNAScan-SE, and RNAmmer predicted 3,406 coding sequences (CDS), 56 tRNA genes, and 4 rRNA genes. 26 copies of insertion sequences were present: 18 of IS*3*, 3 of IS*256*, 1 of IS*481*, 1 of IS*5*, 1 of IS*As1*, 1 of IS*1*, and 1 of IS*200*. The known virulence determinants *hly*, *fim*, *fli*, and an O-antigen gene cluster were also present. The availability of additional sequences, along with an in-depth comparative genome analysis, may help in the identification of those virulence markers responsible for the vaccine failure events produced by this emerging pathogen.

### Nucleotide sequence accession numbers.

The 37551 sequence has been deposited at DDBJ/EMBL/GenBank under the accession no. JPFO00000000. The version described in this paper is the first version, JPFO01000000.
